# Transgastric Migration of a Gossypiboma After an Open Cholecystectomy: A Case Report

**DOI:** 10.7759/cureus.86449

**Published:** 2025-06-20

**Authors:** Sofia Jehanzeb, Bashir Khan, Saad Salman, Aurangzeb Khan, Babar Ali, Muhammad Haseeb Shah, Sumaira Noureen

**Affiliations:** 1 Department of General Surgery, Mardan Medical Complex, Mardan, PAK; 2 Department of Radiology, Mardan Medical Complex, Mardan, PAK

**Keywords:** anorexia, cholesystectomy, gossypiboma, laprotomy, radiological

## Abstract

Gossypiboma refers to the retention of a surgical sponge or pack in a patient's body, postoperatively, with its occurrence most commonly seen intra-abdominally. It is rare but can lead to serious complications if not treated. It can manifest immediately after surgery or can take several years. Clinical presentation can vary from abdominal symptoms to abscess and sepsis. Its diagnosis can be confirmed via radiological modalities such as computed tomography (CT), plain X-ray, ultrasound, and magnetic resonance imaging, with CT being the gold standard. Once confirmed, it should be removed via open or laparoscopic surgery. We present a case of a 35-year-old patient with no known comorbidities who presented to our surgical department with abdominal pain and vomiting, but was vitally stable. Symptoms were persistent with no response to medications. It was found that she had an open cholecystectomy done five months back. CT confirmed the presence of gossypiboma that had eroded the stomach wall and had completely migrated to the stomach, following which an open surgery was performed, and a Promed pack was retrieved from the stomach.

Gossypiboma is a rarely reported error, but it can be a life-threatening complication of surgery. The most common site of occurrence is the abdominal cavity; other sites include the breast, thorax, and central nervous system. It is most commonly associated with cholecystectomy and is most often found in the subhepatic region; however, it can erode the surrounding viscera and migrate to other organs, as seen in our case. Various radiological modalities such as CT, ultrasound, and magnetic resonance imaging can be used to confirm the diagnosis. As gossypiboma is a serious complication, all measures should be taken to prevent it, as its incidence depends on surgical practices. Proper pack counts, using radio-opaque products, and adherence to standard safety protocols can play a vital role in its prevention. Early diagnosis and treatment can prevent this complication and improve patient outcomes.

## Introduction

Gossypiboma is a rare but serious complication seen in surgical patients. It is not usually reported because of medicolegal issues [1]. Gossypiboma has an incidence rate of 1 in 1,000-1,500 in patients undergoing intra-abdominal procedures [2] and 1 in 3,000 in all surgical interventions. The word “Gossypiboma” is derived from the Latin word “Gossypium” that means cotton and a Kiswahili word “Boma” that means area of concealment, and thus it refers to a foreign body made of cotton matrix and surrounded by foreign body granuloma in a patient's body [3]. The first case of gossypiboma reported by Wilson dates back to 1884, when a retained surgical sponge was discovered postoperatively [4]. Gossypibomas are most commonly found in the intra-abdominal cavity; however, they can also be found in the chest [5], extremities [6], central nervous system (CNS) [7], and breast [8]. Gossypiboma can present anytime postoperatively, including immediately or after several months or even decades. It usually presents as a pseudotumor, an occlusive syndrome, or a septic syndrome [9]. It is most common in postcholecystectomy patients; however, a common site in postcholecystectomy patients is the subhepatic region [10]. The clinical manifestation of gossypiboma differs based on its location and the body's response. Acute cases present as fistulas or abscesses, while chronic cases manifest as encapsulated masses with nonspecific symptoms [11]. Clinical symptoms of a gossypiboma are variable and can present as abdominal pain, nausea, vomiting, anorexia, weight loss, altered bowel habits, intestinal perforation, abscess, or sepsis [12]. Diagnosis of gossipiboma can be made based on certain factors such as past surgical history and presenting signs and symptoms; relevant investigations and radiological studies such as plain radiography when surgical textile materials have been impregnated with a radio-opaque marker, ultrasonography (USG), computed tomography (CT), magnetic resonance imaging (MRI), and gastrointestinal contrast series have been useful tools in the diagnosis of gossypiboma [13]. After diagnosis, it is necessary to promptly treat gossypiboma to prevent future complications because the longer the gossypiboma stays in the body, the higher the risk of fistulization [13,14]. Once diagnosed, a gossypiboma should be removed by open or laparoscopic surgery [15]. This case highlights a rare intragastric gossypiboma causing gastric outlet obstruction five months after surgery, underscoring the diagnostic challenge and need for vigilance in postoperative patients.

## Case presentation

In January 2025, a 35-year-old female patient presented to the surgical outpatient department with chief complaints of epigastric pain that was dull, non-radiating, and of moderate intensity, nausea/vomiting, and anorexia. She had been experiencing these symptoms for the past three to four months with no response to medications. On arrival, the patient was vitally stable (BP 110/80 mmHg, HR 76 bpm, SpO_^2^_ 97%, temperature 36.5 °C). She had no known comorbidities. Her surgical history was taken, and a detailed examination was done. On general physical examination, she was pale, ill-looking, dehydrated, and emaciated. On abdominal examination, the abdomen was soft and tender in the epigastrium. Lab investigations revealed a mildly low Hb level that was continuously monitored (Table 1).

**Table 1:** Lab investigations

Investigation	Result	Normal range (approx.)
CBC		
Total leukocyte count (TLC)	8.9 × 10⁹/L	4 – 11 × 10⁹/L
Hemoglobin (Hb)	10.6 g/dL	12 – 16 g/dL
Platelets (Plt)	308 × 10⁹/L	150 – 400 × 10⁹/L
Liver function tests (LFTs)		
Alanine aminotransferase (ALT)	32 U/L	7 – 56 U/L
Aspartate aminotransferase (AST)	28 U/L	10 – 40 U/L
Alkaline phosphatase (ALP)	98 IU/L	44 – 147 IU/L
Total bilirubin	0.8 mg/dL	0.3 – 1.2 mg/dL
Direct bilirubin	0.3 mg/dL	0.1 – 0.4 mg/dL
Albumin	4.2 g/dL	3.4 – 5.4 g/dL
Renal function tests (RFTs)		
Serum creatinine	0.9 mg/dL	0.6 – 1.3 mg/dL
Blood urea nitrogen (BUN)	14 mg/dL	7 – 20 mg/dL
Serum electrolytes		
Potassium (K⁺)	2.42 mmol/L	3.5 – 5.0 mmol/L
Sodium (Na⁺)	136.2 mmol/L	135 – 145 mmol/L
Chloride (Cl⁻)	93.8 mmol/L	98 – 107 mmol/L

**Figure 1:**
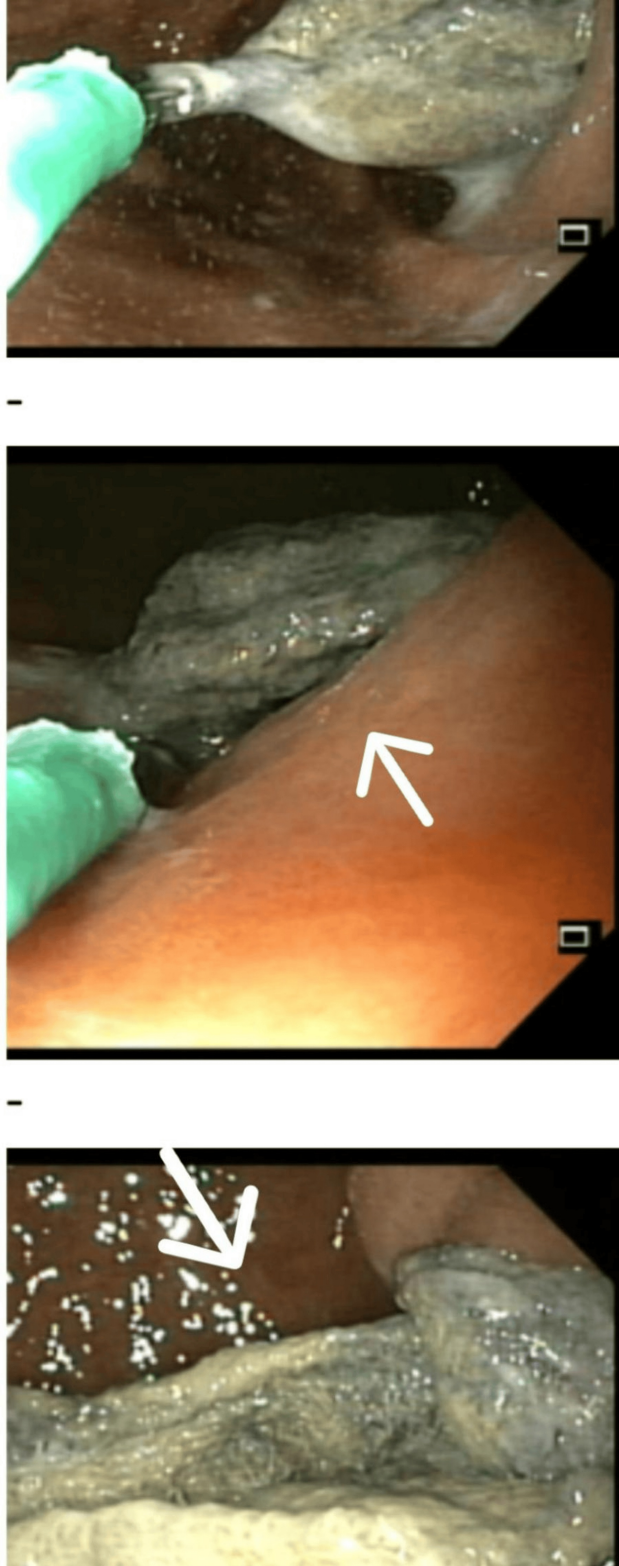
Endoscopic view of the surgical pack stuck in the antrum and pylorus of the stomach

Complete blood count showed a total leukocyte count of 8.9 × 10⁹/L, hemoglobin 10.6 g/dL, and platelets at 308 × 10⁹/L. Liver function tests and renal function tests were normal. Serum electrolytes showed a low serum potassium level at 2.42 mmol/L, and hence, KCl was added to her drug chart; Na was 136.2 mmol/L and Cl 93.8 mmol/L. Endoscopy was done that showed a large abdominal pack stuck in the antrum and pylorus, that couldn't be traversed with the scope and couldn’t be retrieved endoscopically (Figure 1).

**Figure 2:**
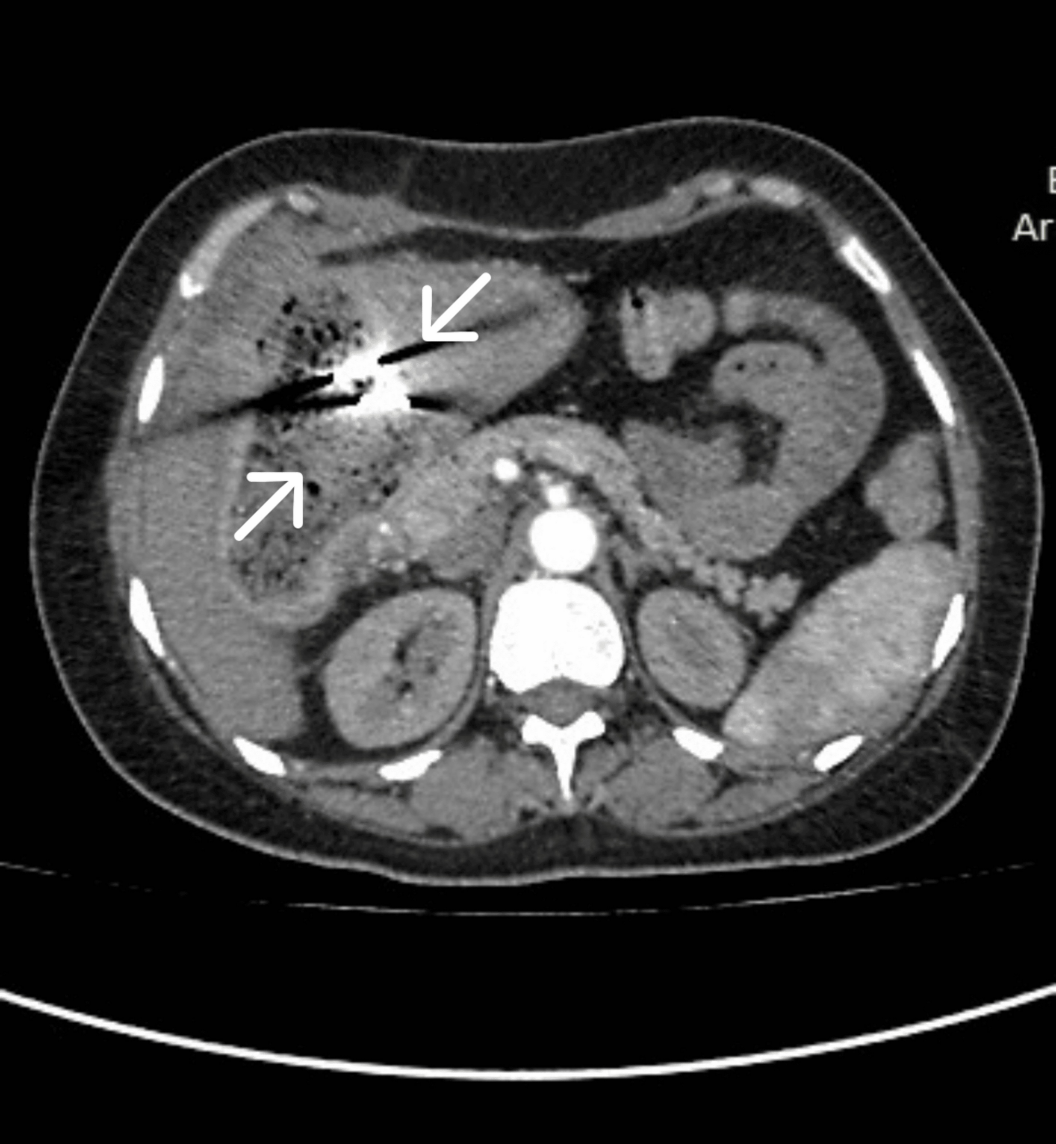
CT (axial view) of the abdomen and pelvis showing the retained surgical pack (arrows)

**Figure 3:**
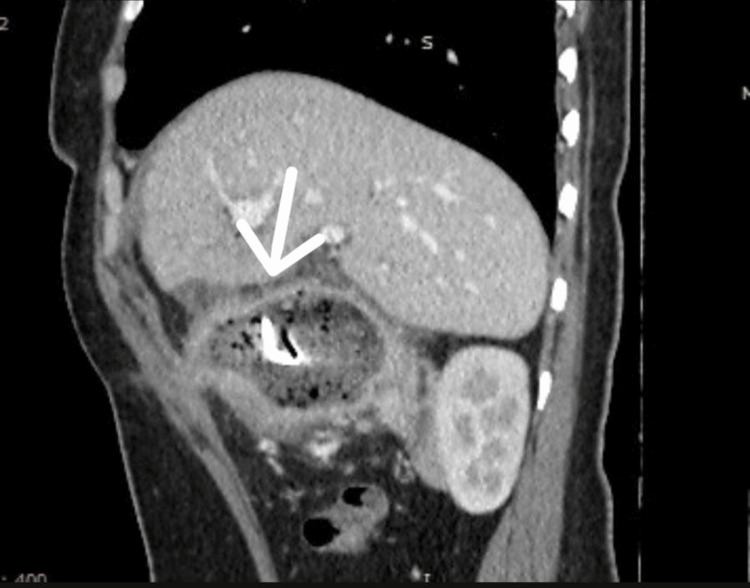
CT (sagittal view) of the abdomen and pelvis showing the retained surgical pack (arrow)

**Figure 4:**
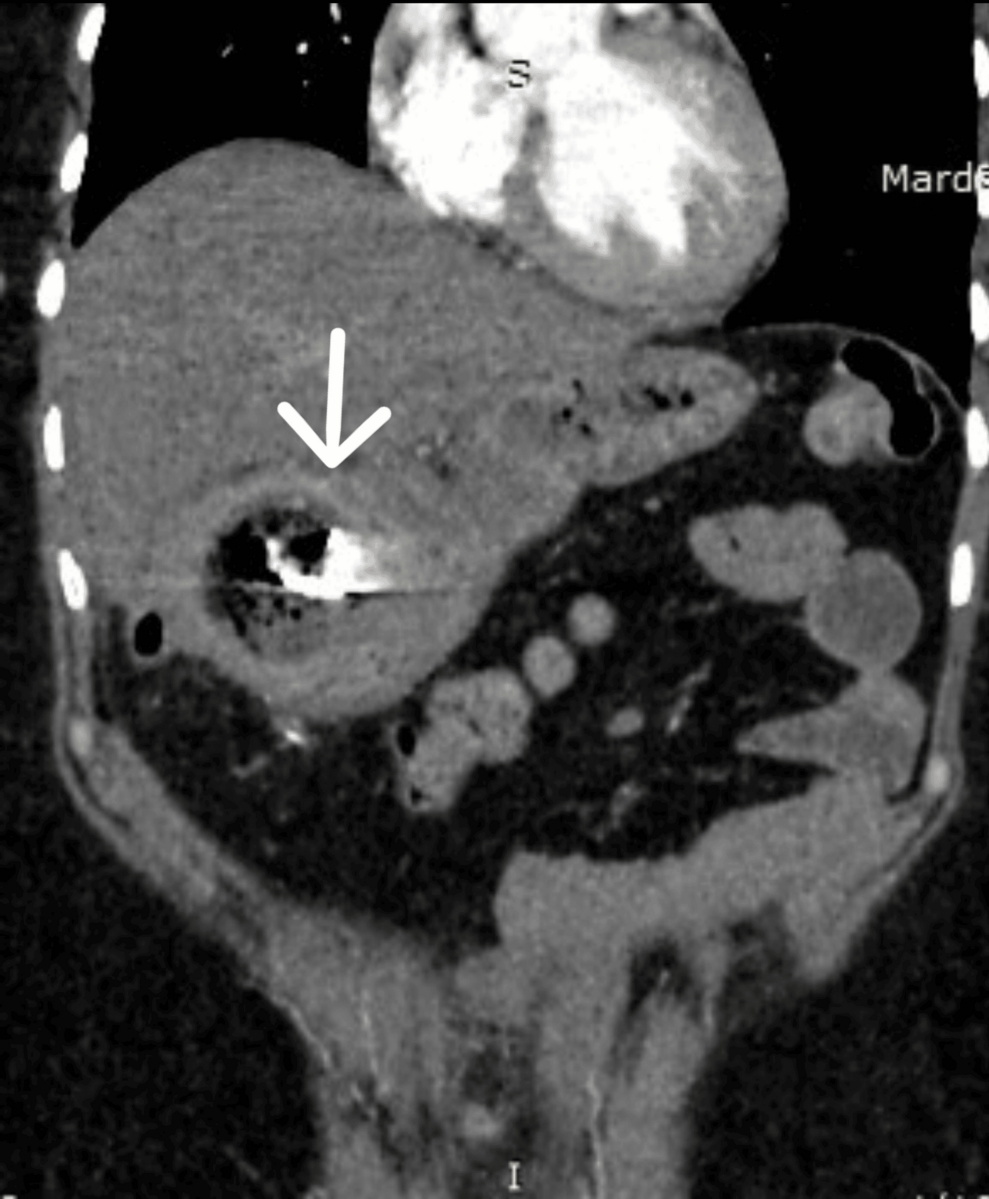
CT (coronal view) of the abdomen and pelvis showing the retained surgical pack (arrow)

CT of the abdomen and pelvis revealed the postcholecystectomy status of mottled lucencies in the distal stomach with an internal dense focus giving streak artifacts closely abutting the stomach wall with surrounding moderate inflammatory changes likely suggesting a foreign body consistent with a gossypiboma (Figures 2-4).

​​She was started on IV fluids and IV medication. Informed written consent was obtained. The patient was optimized for surgery with all the baseline investigations repeated and virology tests done. Subsequently, she was scheduled for open laparotomy. After induction of general anesthesia, the abdomen was prepped and draped in a sterile manner. A 12-cm midline vertical upper abdominal incision was made to enter the peritoneal cavity. Significant adhesions were encountered between the stomach, greater omentum, and the surrounding parietal peritoneum that were carefully released to expose the stomach. A transverse gastrotomy was made over the body of the stomach; a Promed abdominal pack was found loosely adhering to the distal stomach, partially obstructing the pylorus. The pack was soaked with pus, and approximately 15 ml of pus mixed with gastric contents was noted in the area; the foreign body (gossypiboma) was carefully removed. The stomach was thoroughly irrigated with normal saline. The gastrotomy was repaired in a double-layered fashion using Vicryl 2-0 for the inner mucosal layer and seromuscular Lembert sutures for the outer layer. The abdominal cavity was lavaged and a drain was placed.

Post-surgery, she was stable and was kept nil by mouth for three days; after that, oral sips were allowed that she tolerated, so her feed was resumed. She was kept under observation for five days and was discharged. It was decided to proceed with oral treatment on an outpatient basis. She was prescribed oral metronidazole (500 mg every eight hours) and clarithromycin (500 mg once daily) for 14 days. At the second postoperative appointment, the patient remained asymptomatic. A follow-up abdominal ultrasound revealed no evidence of fluid collection or abnormalities.

## Discussion

A retained surgical pack or sponge, although not common, can cause serious complications such as GI obstruction, fistula, GI bleeding, and abscess formation [1]. The lack of a national or local register and the reluctance of medical institutions to publish matters that may have medicolegal implications result in underreporting of such cases [16]. It can present anytime postoperatively, either immediately or years after the initial surgery [3]. The affected body shows two types of responses to gossypiboma: in one, an abscess with or without bacterial infection can form, and the other is an aseptic fibrinous response, which results in tissue adhesions and encapsulation and, eventually, the formation of a foreign body granuloma [3]. The patient may or may not have symptoms, and the symptoms can be acute or sub-acute. Patients present with complaints of abdominal pain, nausea, vomiting, anorexia, weight loss, or a malabsorption-type syndrome [17]. The associated complications include adhesions, abscess formation, intraluminal bacterial overgrowth, fistula, obstruction, and transmural migration into the gastrointestinal tract or adjacent viscera [18]. The literature shows that hepatic gossypiboma is associated with bile duct obstruction and jaundice [19]. Gossypiboma in the gastrointestinal system is associated with cholecystectomy (most common), followed by c-section, hysterectomy, laparotomy, nephrectomy, splenectomy, hemicolectomy, distal gastrectomy, appendectomy, anterior resection of the hydatid cyst, cystectomy, and myomectomy [16]. Various radiological modalities can be used to diagnose gossypiboma, such as ultrasound, CT, MRI, and X-ray. However, all can be inconclusive if the pack does not have a radiological marker and can be misdiagnosed as hematoma, abscess, or neoplasm [10,20]. Once confirmed, the mainstay of treatment for gossypiboma is open or laparoscopic surgery. Laparoscopic surgery is the choice of modality because of less pain, shorter hospital stays, smaller incisions, lower infection rates, and reduced hemorrhage compared to open surgery [15]. However, other modalities can also be used, such as endoscopic procedures, if it lies entirely in the gastrointestinal tract [13]. As we know, prevention is better than cure. Human errors can’t be completely omitted, but good medical training and adherence to standard safety rules in the operation theater can reduce the incidence of such cases to a minimum [14].

## Conclusions

The key feature of our case was the complete migration of the surgical pack into the stomach postcholecystectomy in five months. Therefore, it is recommended to consider gossypiboma in patients presenting with abdominal pain and a history of previous surgery. This report adds to the existing evidence of postcholecystectomy gossypiboma, highlighting the importance of practicing preventive measures to stop it from happening and early diagnosis to prevent complications from occurring. Although it is rare, it can be a life-threatening complication of all surgical procedures. Hence, all measures should be taken to prevent this, as its incidence depends on surgical practices. Proper pack counts, using radio-opaque products, adherence to standard safety protocols, final cavity inspection, and team communication during surgery can play a vital role in the prevention of the occurrence of gossypiboma. Prompt diagnosis and early treatment can prevent complications and improve patient outcomes.
